# Nipah Virus Impairs Autocrine IFN Signaling by Sequestering STAT1 and STAT2 into Inclusion Bodies

**DOI:** 10.3390/v15020554

**Published:** 2023-02-17

**Authors:** Nico Becker, Andrea Maisner

**Affiliations:** Institute of Virology, Philipps University Marburg, Hans-Meerwein-Str. 2, 35043 Marburg, Germany

**Keywords:** Nipah virus, phosphoprotein, inclusions, STAT1, STAT2, IFN, autocrine signaling

## Abstract

Nipah virus (NiV) is an emerging zoonotic paramyxovirus that causes fatal infections in humans. As with most disease-causing viruses, the pathogenic potential of NiV is linked to its ability to block antiviral responses, e.g., by antagonizing IFN signaling through blocking STAT proteins. One of the STAT1/2-binding proteins of NiV is the phosphoprotein (P), but its functional role in IFN antagonism in a full viral context is not well defined. As NiV P is required for genome replication and specifically accumulates in cytosolic inclusion bodies (IBs) of infected cells, we hypothesized that this compartmentalization might play a role in P-mediated IFN antagonism. Supporting this notion, we show here that NiV can inhibit IFN-dependent antiviral signaling via a NiV P-dependent sequestration of STAT1 and STAT2 into viral IBs. Consequently, the phosphorylation/activation and nuclear translocation of STAT proteins in response to IFN is limited, as indicated by the lack of nuclear pSTAT in NiV-infected cells. Blocking autocrine IFN signaling by sequestering STAT proteins in IBs is a not yet described mechanism by which NiV could block antiviral gene expression and provides the first evidence that cytosolic NiV IBs may play a functional role in IFN antagonism.

## 1. Introduction

Nipah virus (NiV) is an emerging, zoonotic and highly pathogenic RNA virus of the family *Paramyoxviridae*, order *Mononegavirales* (MNS). Since its discovery in 1998, NiV has caused sporadic outbreaks of disease in South and Southeast Asia (Malaysia, Singapore, Bangladesh, India) [1,2]. Fruit bats are the primary reservoir for NiV, from which the virus has been transmitted to humans either via pigs as intermediate hosts or via virus-contaminated food or liquids. In many outbreaks, NiV was also transmitted from person to person [3,4,5]. Clinical human cases usually show symptoms of acute encephalitis and respiratory illness, with case fatality rates of at least 40% [6]. As there are no vaccines or specific antiviral drugs available, NiV and henipaviral diseases have been identified as a priority disease for the WHO Research and Development Blueprint [7].

NiV is an enveloped, negative-sense, non-segmented, single-stranded RNA virus and encodes six structural proteins. The N protein (nucleoprotein), the P protein (phosphoprotein) and the large L protein (polymerase) encapsidate the viral RNA and form the viral ribonucleoprotein complex (RNP). The NiV M (matrix) protein plays a central role in virus assembly [8] and interacts with the RNP and the two NiV surface glycoproteins G (glycoprotein) and F (fusion protein) [9]. During virus entry, the NiV G protein binds to ephrin-B2/-B3 receptors on the host cell [10,11,12], while the F protein mediates the fusion of the viral envelope and the plasma membrane [13,14]. NiV G/F glycoprotein complexes expressed on the surfaces of infected cells also mediate cell-to-cell fusion with neighboring non-infected cells, which facilitates the spread of infection and typically results in the formation of multinucleated cells or syncytia [15].

NiV-infected cells generally contain inclusion bodies (IBs), both in cell cultures and in histopathological specimens of Nipah patients or infected animals [16,17,18,19,20]. Such cytosolic IBs are a general hallmark of MNS infections and are therefore consistently found in paramyxovirus-infected cells. As membrane-less viral compartments or biomolecular condensates, IBs are assembled by liquid–liquid phase separation and are highly enriched in nucleocapsid proteins [21,22,23,24,25,26,27]. In NiV-infected cells, cytosolic IBs are rapidly formed since only the highly abundant NiV N and P proteins are required for their formation [20]. Similar to IBs of other MNS, cytosolic NiV IBs likely play a role in viral replication, although their involvement in viral RNA synthesis or RNP formation has not been well defined. In addition, they certainly have other functions. For example, we recently showed that NiV IBs have some aggresome-like properties by recruiting unrelated, overexpressed or nonfunctional cytosolic proteins, thereby reducing potential proteotoxic stress in an infected cell [28]. On top of this, NiV IBs likely play a role in inhibiting the antiviral interferon response in infected host cells, as we will demonstrate in the study presented here. 

Interferons (IFNs) are the most important innate antiviral cytokines and virus-induced IFN responses comprise two phases, IFN induction and IFN signaling. In the induction phase, viral RNA stimulates the expression and secretion of type I IFNs (especially IFN-α/β) and type III IFNs (IFN-λ). IFNs secreted from infected cells bind to their respective receptors expressed either on the infected cell itself, inducing autocrine IFN signaling, or on non-infected neighboring cells triggering paracrine IFN signaling [29]. Although type I and III IFNs use different cell surface receptors (IFNAR1/2 and IFNLR1/IL-10R2), they share similar downstream signaling pathways mediated by Janus kinases (JAK). These then phosphorylate and activate the signal transducer and activator of transcription 1 (STAT1) and 2 (STAT2), which finally induce a broad range of IFN-stimulated genes (ISGs) [30]. Virtually all pathogenic RNA viruses have evolved strategies to evade antiviral innate immune responses [31,32]. This also applies to paramyxoviruses, which have developed multiple highly varying mechanisms to block IFN induction or IFN signaling, with STAT1 and STAT2 as the primary targets of IFN-antagonistic viral proteins [33,34,35,36]. NiV also inhibits STAT activation, and the NiV P gene products are known to be primarily responsible for this IFN signaling blockade (for a review, see [37]). The full-length P protein, as well as the two nonstructural accessory NiV proteins V and W, which are produced as a result of P mRNA editing, bind STAT1 and STAT2 via a shared N-terminal region with two distinct STAT binding sites. Consequently, the NiV P, V and W proteins can each inhibit the IFN-dependent phosphorylation/activation of STAT proteins [38,39]. While the NiV P and V proteins act in the cytoplasm, the nuclear W protein binds STAT1 after it enters the nucleus and prevents it from shuttling back to the cytoplasm [40,41].

The accessory NiV proteins have been studied intensively in vitro [38,39,40,41,42] and in vivo [43,44,45], but the functional and mechanistic role of the full-length P protein is not well characterized. Data on the interactions of NiV P with STAT proteins come almost exclusively from transiently expressed P proteins in the absence of other NiV proteins [38,39,46]. In a viral context, however, the P protein is always bound to NiV N proteins, which leads to the formation of cytosolic IBs. The fact that P accumulates in IBs in NiV-infected cells led us to hypothesize that IBs may play a functional role in the NiV P-mediated inhibition of IFN signaling by recruiting STAT1 and STAT2. Substantiating this idea, we show here that STAT1 and STAT2 massively accumulate in IBs, both in NiV-infected cells and in cells co-expressing only NiV N and P proteins, which is the minimal requirement for IB formation [20,47]. We further demonstrate that STAT sequestration to IBs prevents IFN-dependent pSTAT1 and pSTAT2 formation and translocation to the nucleus, thereby blocking autocrine IFN signaling in infected cells. By this, we show for the first time that cytosolic NiV IBs play a functional role in IFN antagonism.

## 2. Materials and Methods

### 2.1. Virus Infection

The NiV_Malaysia_ (NiV) isolate used in this study has been described previously [48]. All infection experiments with NiV were performed under BSL-4 conditions at the Institute of Virology, Philipps University Marburg, Germany. A549 cells (human lung epithelial cells, ATCC CCL-185) were cultivated in Dulbecco’s modified Eagle’s medium (DMEM, Gibco–ThermoFisher, Waltham, MA, USA) containing 10% fetal calf serum (FCS, Gibco), 100 U penicillin ml^−1^ (Gibco), 0.1 mg streptomycin mL^−1^ (Gibco) and 4 mM L-glutamine (Gibco). Confluent A549 cells grown either on coverslips (3.75 × 10^5^ cells) or on 6-wells (1 × 10^6^ cells) were infected with NiV at a multiplicity of infection (MOI) of 10, basically as described earlier [48]. After virus infection for 1 h at 37 °C, cells were washed five times with DMEM 2% FCS and then incubated in DMEM 2% FCS at 37 °C for 24 or 48 h. For additional stimulation with exogenous IFN-β, NiV-infected cells were incubated with DMEM without FCS for 60 min. Subsequently, cells were treated with DMEM containing 1000 U/mL human interferon-beta 1b (Prospec, Ness-Ziona, Israel) for 40 min before fixation.

### 2.2. Plasmids and Transfection

pCG-vector based expression plasmids encoding NiV N, NiV P and NiV PeGFP have been described previously [20,28]. The STAT1-binding defective NiV P_G125E_ mutant has been described earlier by Ciancanelli et al., 2009 [41]. A549 cells grown on coverslips to 80% confluence were transfected with a total of 1 µg of DNA (equal amounts of pCG-N and pCG-PeGFP plasmids) using Lipofectamine 2000 (Invitrogen-ThermoFisher, Waltham, MA, USA), according to the manufacturer’s protocol. At 4 h post-transfection, the medium was replaced, and the cells were incubated in DMEM 10% FCS at 37 °C for 22–24 h. For IFN stimulation, transfected cells were first incubated with DMEM without FCS for 60 min and then with DMEM containing 1000 U/mL human interferon-beta 1b (Prospec) for 40 min before fixation.

### 2.3. Confocal Immunofluorescence Analysis

Immunostaining was performed as described previously [28]. Transfected cells were fixed with 4% paraformaldehyde (PFA, Merck, Darmstadt, Germany) in DMEM. PFA was quenched by 0.1 M glycine (Carl Roth, Karlsruhe, Germany) in phosphate-buffered saline (PBS, ThermoFisher, Waltham, MA, USA) supplemented with MgCl_2_ (Merck) and CaCl_2_ (Merck) (designated as PBS++). Cells were permeabilized with 0.1% Triton X-100 (Merck) or ice-cold methanol (Sigma-Aldrich–Merck, Darmstadt, Germany) and treated with a blocking buffer containing 2% BSA (Serva, Heidelberg, Germany), 5% glycerol (Carl Roth), 0.2% Tween20 (Sigma-Aldrich), 0.05% NaN_3_ (Merck). For primary antibody incubation, coverslips were removed from the 24-well plate, inverted and placed on a 20 μL drop of the respective primary antibody solution in blocking buffer applied on a parafilm (cells facing down). After incubation for 1 h at 4 °C, coverslips were placed back in a 24-well plate (cells facing up) and washed three times with PBS++, before adding 300 µL of the appropriate Alexa Fluor-conjugated secondary antibody. A list of the used primary and secondary antibodies, as well as the respective dilutions and permeabilization method, is provided in the Supplementary Materials (Table S1).

Immunostaining of endogenous cellular proteins in NiV-infected cells was performed within the BSL-4 laboratory. Samples were then inactivated with 4% PFA for 48 h. After removal from the high containment laboratory, PFA was quenched with 0.1 M glycine in PBS++. To visualize NiV-positive cells and viral IBs, NiV N and P proteins were stained with a polyclonal guinea pig NiV antiserum [49] and Alexa Fluor 488-conjugated secondary antibodies (Table S1), as described above. Nuclei were counterstained with 4′,6-diamidino-2-phenylindole (DAPI). The coverslips were then mounted with mowiol (Calbiochem–Merck, Darmstadt, Germany) and analyzed using a confocal laser scanning microscope (Leica TCS SP5 II).

### 2.4. Image Analysis to Quantify Nuclear Translocation of STATs

To calculate the relative nuclear STAT fluorescence, confocal fluorescence images were captured using sequential acquisition to obtain separate image files for each channel (DAPI, eGFP and Alexa Fluor 568). Signal quantification was performed using the ImageJ software (http://rsb.info.nih.gov/ij, accessed on 9 January 2023). To define regions of interest (ROIs), whole cells were manually traced using the ImageJ area selection tool (ROI-1), and a nuclear mask was created using the DAPI channel, Huang’s thresholding method and the particle analyzer tool (ROI-2). STAT signal intensities in ROI-1 (total cellular STAT) and signal intensities in ROI-2 (nuclear STAT) were measured and presented as “relative nuclear fluorescence” (ratio of nuclear to total cellular fluorescence) after background subtraction. For both STAT1 and STAT2, at least 300 cells were analyzed. Only cells that met the following criteria were included in the analysis: well-defined cell borders and intact nuclei. If cells contained NiV IBs, these had to be at least medium in size. Results are presented as mean ± SD, and statistical comparison was performed using an unpaired *t*-test.

### 2.5. Image Analysis to Quantify Nuclear pSTAT Levels

To determine the nuclear pSTAT1 fluorescence, confocal fluorescence image files for each channel (DAPI, Alexa Fluor 488 and Alexa Fluor 568) were analyzed with the ImageJ software. Nuclei were selected by creating a nuclear mask using the DAPI channel, Huang’s thresholding method and the particle analyzer tool. The pSTAT1 signal intensity was measured and presented as “mean nuclear fluorescence” (sum of the nuclear signal intensity divided by the nuclear area) after background subtraction. In total, over 800 cells were evaluated. Results are presented as mean ± SD, and statistical comparison was performed with the GraphPad PRISM software using a Kruskal–Wallis test, followed by Dunn’s multiple comparisons test.

### 2.6. Quantitative Real-Time Polymerase Chain Reaction

Quantitative PCR (qPCR) analysis was performed as described previously [50,51]. Total RNA was isolated from lysates of infected cells (RNeasy Kit, Qiagen, Venlo, Netherlands) and reverse-transcribed with oligo(dT)_18_ primers (RevertAid First Strand cDNA Synthesis Kit, ThermoFisher). qPCR was performed in technical quadruplicates using Maxima SYBR Green qPCR Master Mix (2X, ThermoFisher), 50 ng of cDNA and 60 pmol of the respective forward and reverse primers. Primer pair sequences are provided in the Supplementary Materials (Table S2). Amplification was carried out using a StepOne Real-Time PCR System (Applied Biosystems–ThermoFisher, Waltham, MA, USA) with the following cycling conditions: initial denaturation at 95°C for 10 min, followed by 40 cycles of amplification (denaturation at 95 °C for 15 s, annealing at 53 °C for 15 s, extension at 72 °C for 30 s). Ct values of NiV-infected and non-infected (mock) samples were normalized by subtracting the Ct value of α-tubulin (ΔCt), which was used as a reference housekeeping gene. To calculate the changes in mRNA levels in NiV-infected cells (fold change over mock), the ΔCt values of the respective mock samples were subtracted (ΔΔCt) and represented using the 2^−ΔΔct^ method [52]. qPCR results of three individual experiments are presented as mean ± SD.

## 3. Results

### 3.1. STAT1 and STAT2 Are Sequestered to Cytosolic IBs in NiV-Infected Cells

**Figure 1:**
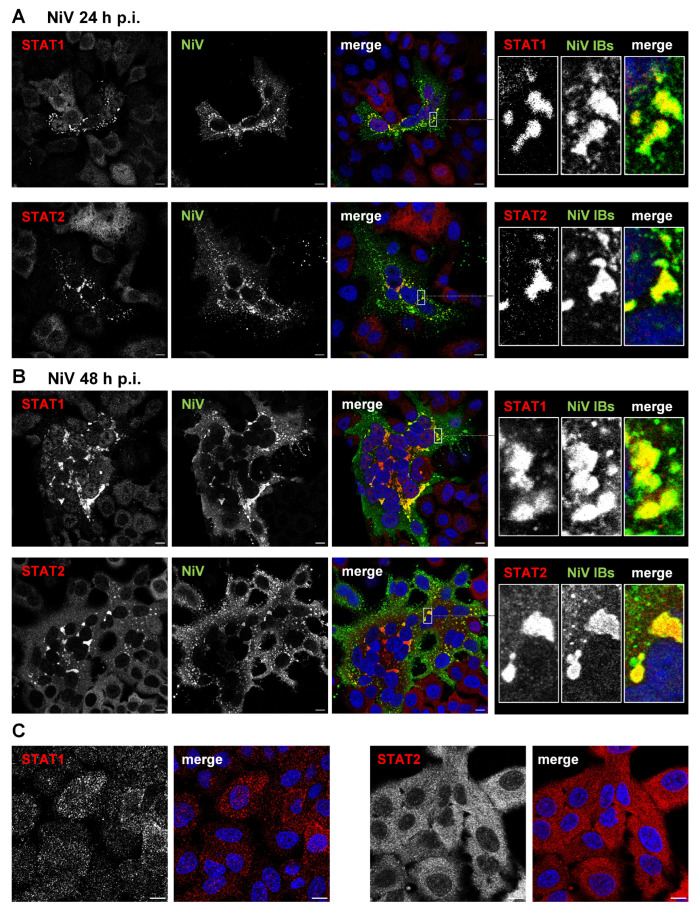
STAT1 and STAT2 accumulate in cytosolic IBs formed in NiV-infected cells. A549 cells were infected with NiV at an MOI of 10. At 24 h p.i. (**A**) or 48 h p.i. (**B**), cells were fixed with PFA and permeabilized with Triton X-100. Endogenous STAT1 and STAT2 were stained with specific antibodies (red) and NiV-positive syncytia with IBs were visualized with a NiV N/P specific antiserum (green). Nuclei were counterstained with DAPI (blue). The single-channel greyscale images of the labeled proteins as well as the colorized merged images are shown. Boxed areas are displayed in a higher magnification in the right panels. (**C**) STAT1 and STAT2 distribution in non-infected A549 cells is shown. Scale bars, 10 µm.

A hallmark of NiV infection is the formation of intracellular inclusions, in which viral nucleocapsid proteins are concentrated. Thus, the P protein in NiV-infected or in NiV N/P coexpressing cells always accumulates in cytosolic IBs. This raised the question of whether the accumulation of P proteins in IBs prevents cytosolic interactions with STAT1 and STAT2 or whether STAT proteins are sequestered into IBs along with P proteins, altering the usually dispersed steady-state distribution of STAT1/2. To address this, we analyzed the intracellular localization of STAT1 and STAT2 in NiV-infected cells. At either 24 h or 48 h post infection (p.i.), A549 cells infected with NiV were fixed and permeabilized. To label infected cells and visualize viral IBs, cells were immunostained with NiV N/P-specific polyclonal antibodies. Endogenous STAT1 and STAT2 proteins were detected with respective monoclonal antibodies. As expected, small NiV syncytia had formed at 24 h p.i. (Figure 1A), while larger syncytia were found at 48 h p.i. (Figure 1B). At both time points, numerous cytosolic NiV IBs were evident and these clearly colocalized with STAT1 and STAT2 (Figure 1, boxed areas). While both STAT proteins accumulated in the IBs of infected cells, they were homogenously distributed in non-infected cells (Figure 1C). This clearly supports the idea that STAT proteins are specifically recruited to IBs.

**Figure 2:**
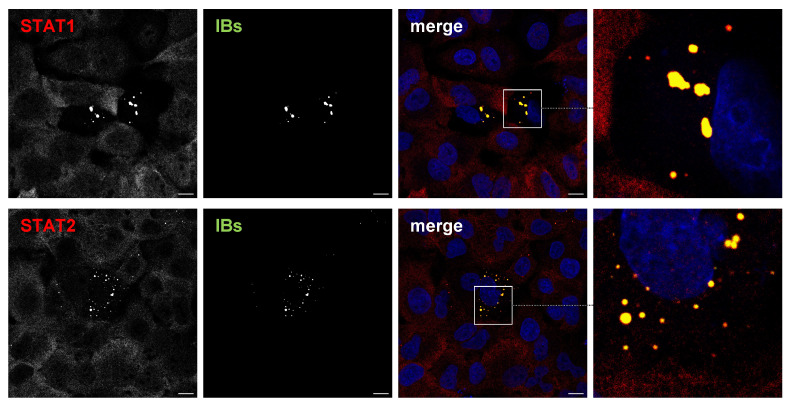
NiV N/P inclusions sequester STAT proteins. A549 cells were transfected with plasmids encoding NiV N and NiV PeGFP, the minimal requirement to induce the formation of cytosolic IBs. After 24 h, cells were fixed with PFA, permeabilized with Triton X-100 and endogenous STAT1, and STAT2 proteins were stained with specific antibodies (red). Inclusion bodies (IBs) were detected by eGFP autofluorescence (green). Nuclei were counterstained with DAPI (blue). Greyscale single-channel images and colorized merged images are shown. Boxed areas are displayed in a higher magnification and show the STAT accumulation in IBs in a NiV N/P-expressing cell. Scale bars, 10 µm.

Next, we wanted to ensure that STAT recruitment to IBs depends on the NiV P protein and does not require the P gene products W and V. These accessory proteins share the N-terminal STAT1 and STAT2 binding sites with the full-length P protein and are expressed in infected cells due to P mRNA editing by the NiV polymerase [53]. To rule out that V or W proteins are involved, we analyzed the STAT1/2 distribution in transfected cells expressing only NiV N and P proteins, the minimal requirement for IB formation [20,47]. In A549 cells cotransfected with plasmids encoding the NiV N and a GFP-tagged NiV P protein, IBs had readily formed after 24 h (Figure 2). Similar to infected cells, STAT1 and STAT2 accumulated in these cytosolic IBs, demonstrating that neither viral replication nor any of the nonstructural P gene products are required for STAT sequestration to IBs.

### 3.2. Recruitment to IBs Blocks IFN-Triggered Nuclear Accumulation of STAT1 and STAT2

**Figure 3:**
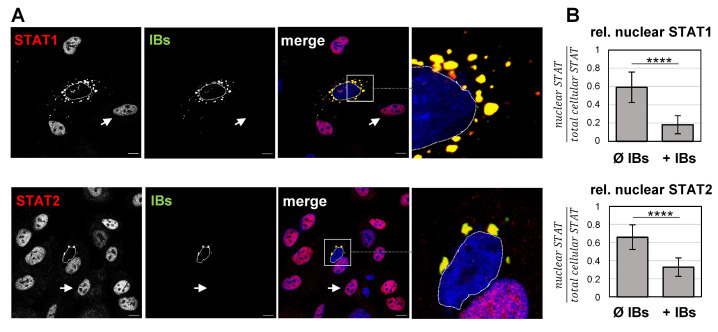
NiV IBs prevent nuclear translocation of STAT proteins after IFN treatment. (**A**) A549 cells were transfected with plasmids encoding NiV N and NiV PeGFP for 22 h to induce IB formation. Then, cells were treated with 1000 U/mL of human IFN-β for 40 min and fixed with PFA. After permeabilization with Triton X-100, endogenous STAT1 and STAT2 proteins were stained with specific antibodies (red). IBs were detected by eGFP autofluorescence (green). Nuclei were counterstained with DAPI (blue). Boxed areas are displayed in a higher magnification and show the STAT accumulation in IBs in a NiV N/P-expressing cell. Arrows point to the nucleus of a non-transfected cell without IBs. The dashed lines label the nuclei of IB-containing cells. Scale bars, 10 µm. (**B**) To quantify the relative STAT fluorescence in the nucleus, the total cellular fluorescence as well as nuclear signal intensities of STAT1 or STAT2 were quantified in at least 300 cells. The ratio of nuclear to total cellular fluorescence in cells without IBs (Ø IBs) and with IBs (+IBs) is shown (relative nuclear STAT). Error bars indicate standard deviation (SD); **** *p* < 0.0001.

To test whether the sequestration of STAT1 and STAT2 in IBs has functional consequences and affects IFN-dependent activation and nuclear STAT accumulation, STAT localization in IFN-treated cells was analyzed. The addition of IFN normally triggers the phosphorylation of IFN receptor-associated JAK kinases (JAK1, TYK2), which, in turn, recruit and phosphorylate STAT1 and STAT2. Activated pSTAT1/pSTAT2 heterodimers, together with the interferon regulatory factor 9 (IRF9), then translocate to the nucleus to induce a broad range of IFN-stimulated genes (ISGs) [30]. To determine if pSTAT translocation into nuclei in response to IFN stimulation is prevented in cells with IBs, A549 cells expressing N and P proteins were treated with IFN-β for 40 min and fixed to analyze the subcellular STAT distribution. As expected, IFN signaling induced STAT1 and STAT2 translocation and accumulation in nuclei of non-transfected cells (Figure 3A, arrows). In contrast, STAT1 and STAT2 were concentrated in IBs and nuclear STAT fluorescence was barely detected in N/P-expressing cells (Figure 3A, boxed and magnified areas). Image quantifications confirmed that the nuclear STAT signals in IB-containing cells were significantly reduced in contrast to non-transfected cells without IBs (Figure 3B).

### 3.3. IFN-Induced Formation of pSTAT Is Prevented in the Presence of IBs

**Figure 4:**
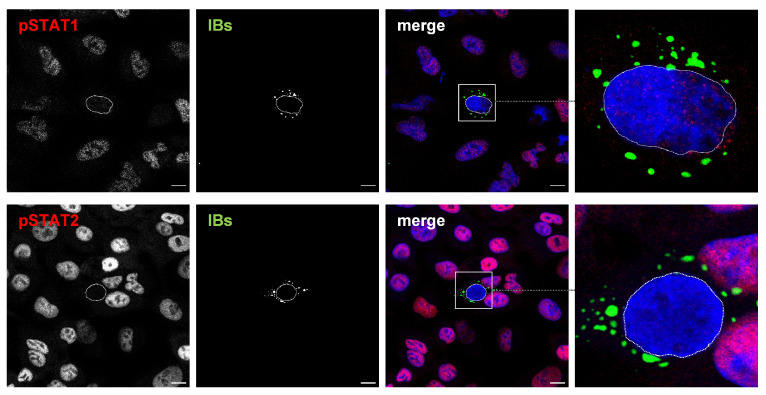
NiV IBs prevent formation of pSTAT in IFN-treated cells. A549 cells were transfected with plasmids encoding NiV N and NiV PeGFP. Then, 22 h after transfection, cells were treated with IFN-β (1000 U/mL) for 40 min. Afterwards, cells were fixed with PFA, permeabilized with methanol at −20 °C, and endogenous pSTAT1 and pSTAT2 were stained with specific antibodies (red). IBs were detected by eGFP autofluorescence (green). Nuclei were counterstained with DAPI (blue). The greyscale image of the labeled proteins as well as the colorized merged images are shown. The dashed lines highlight the nuclei of IB-containing cells. Boxed areas are displayed in a higher magnification. Scale bars, 10 µm.

The absence of nuclear STAT1/2 in cells containing NiV IBs might either be due to the fact that phosphorylated STAT1/STAT2 was not formed upon IFN treatment, or because pSTAT was formed but then sequestered to IBs, preventing its translocation into the nucleus. To discriminate between these two possibilities, we analyzed the pSTAT distribution after IFN stimulation. As shown in Figure 4, pSTAT1 and pSTAT2 were readily detectable in the nuclei of non-transfected cells without IBs. This demonstrates that IFN treatment was functional and had resulted in efficient STAT activation and nuclear pSTAT accumulation. In contrast, N/P-expressing cells with numerous IBs showed hardly any pSTAT signals, neither in the nucleus nor in IBs (Figure 4, boxed areas). This general lack of pSTAT in the presence of IBs suggests that the sequestration of cytosolic STAT1 and STAT2 to IBs completely prevented their phosphorylation by IFN receptor-bound JAK in response to IFN treatment.

### 3.4. NiV Induces IFN and ISG Upregulation in Infected Cell Cultures

**Figure 5:**
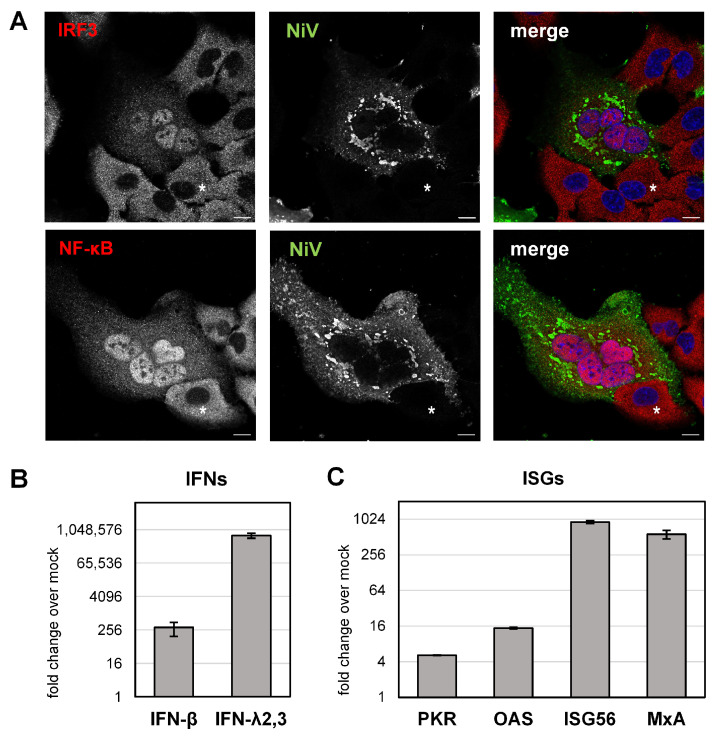
IFN upregulation and ISG expression in NiV-infected cell cultures. (**A**) A549 cells were infected with NiV at an MOI of 10. At 24 h p.i., cells were fixed with PFA, permeabilized with Triton X-100 and IRF3 or NF-κB were stained with specific antibodies (red). NiV-infected cells and IBs were visualized with a NiV N/P-specific antiserum (green). Nuclei were counterstained with DAPI (blue). The single-channel greyscale and the colorized merged images are shown. Asterisks label a non-infected cell adjacent to an infected cell. Scale bars, 10 µm. (**B**,**C**) A549 cells were infected with NiV at an MOI of 10 for 24 h. Then, cells were harvested, and total RNA was isolated and reverse-transcribed using oligo(dT) primers. cDNA was analyzed by quantitative real-time PCR using specific primers for human IFN-β, IFN-λ2,3, PKR, OAS, ISG56 and MxA. Upregulation of IFNs and ISGs is shown as fold change over mock (2^−ΔΔct^). Error bars indicate the standard deviation of three replicate experiments.

To address how STAT1/2 sequestration to IBs functionally affects IFN-mediated antiviral activity in a full viral context, we analyzed IFN induction and ISG expression in NiV-infected cell cultures. In paramyxovirus infections, IFN induction is triggered by viral RNA, which is primarily sensed by cytoplasmic retinoic acid-inducible gene I (RIG-I) or by endosomal Toll-like receptor 3 (TLR3) [36]. These subsequently activate latent cytoplasmic transcription factors known as interferon regulatory factor-3 (IRF3) and nuclear factor κB (NF-κB). After translocation into the nucleus, IRF3 and NF-κB then induce IFN gene transcription [36]. To determine the ability of NiV to induce IFN expression in A549 cells, IRF3 and NF-κB, both essential for IFN induction in these cells [54], were immunostained. At 24 h p.i., both transcription factors were found in nuclei of NiV-positive cells (Figure 5A), demonstrating that NiV had efficiently activated the RIG-I and TLR3 signaling pathways. In contrast to infected cells, IRF3 and NF-κB were homogenously distributed in the cytoplasm of adjacent non-infected cells (Figure 5A, asterisks), showing that the transcription factors were not activated in the absence of viral infection. 

To determine whether viral sensing and nuclear IRF3 and NF-κB accumulation had led to the upregulation of IFNs, total RNA was isolated from infected cell cultures to quantitate IFN gene transcription by qPCR analysis. As the human airway cell line A549 is known to respond to viral infection with the upregulation of type I (IFN-α/β) and type III IFN (IFN-λ) [54], we quantified the IFN-β and IFN-λ expression. As shown in Figure 5B, both IFNs were clearly induced, with IFN-λ being more efficiently upregulated, consistent with our earlier observations in human primary airway epithelial cell cultures [51,55]. IFN induction in NiV-infected cell cultures also led to the upregulation of several ISGs, as exemplified by PKR, OAS, ISG56 and MxA (Figure 5C). 

ISG upregulation in infected cell cultures is probably a result of paracrine IFN signaling in non-infected cells, since autocrine IFN signaling in infected cells is assumed to be prevented by the sequestration of STAT1 and STAT2 to IBs.

### 3.5. Autocrine IFN Signaling Is Blocked in NiV-Infected Cells

**Figure 6:**
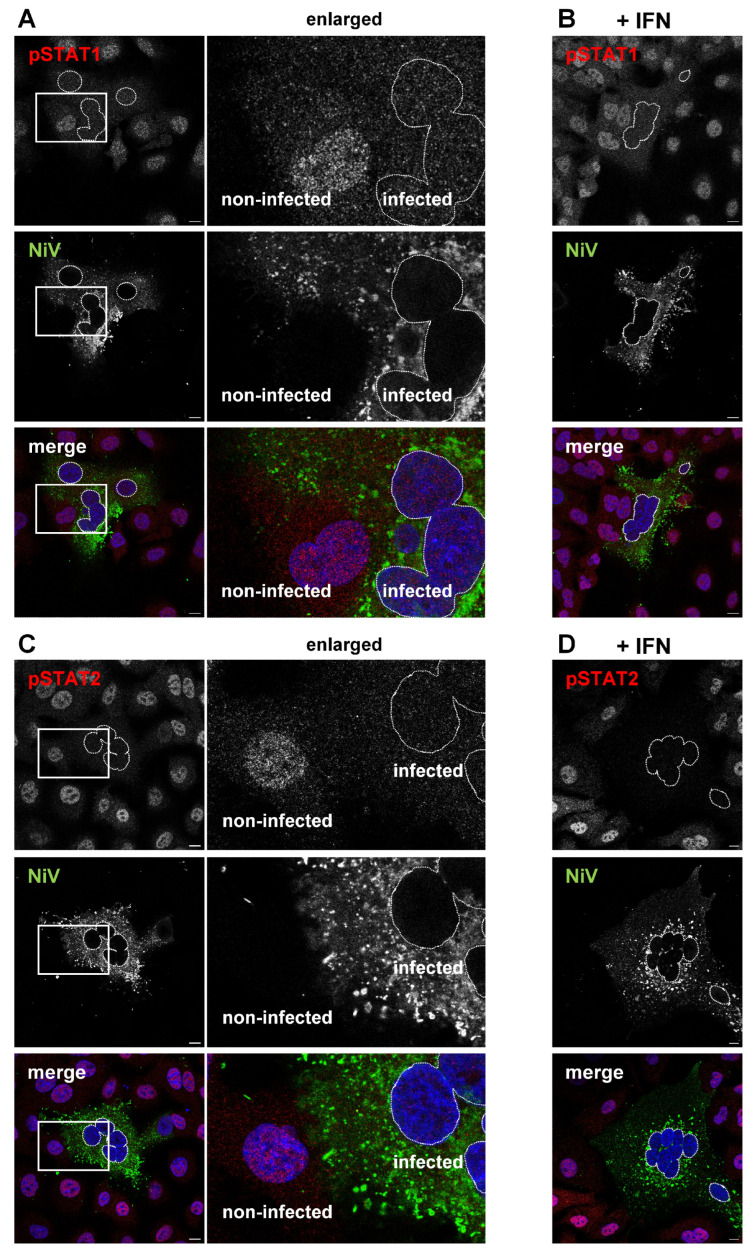
pSTAT formation is prevented in NiV-infected cells. (**A**,**C**) A549 cells were infected with NiV at an MOI of 10 for 24 h, and then fixed with PFA and permeabilized with ice-cold methanol. To monitor autocrine and paracrine IFN signaling induced by IFNs secreted from infected cells, pSTAT1 (**A**) and pSTAT2 (**C**) were detected with specific antibodies (red). NiV-infected cells were labeled with a NiV N/P-specific antiserum (green), and nuclei were counterstained with DAPI (blue). The greyscale images of the pSTAT and the NiV stainings and the colorized merged images are shown. The dashed lines label the nuclei of infected cells. The boxed areas are displayed enlarged in the middle panel and highlight a nucleus in an infected and an adjacent non-infected cell. (**B**,**D**) Cells infected with NiV for 24 h were treated with 1000 U/mL of human IFN-β and fixed after 40 min. Nuclear pSTAT1 (**B**) and pSTAT2 (**D**) in non-infected and in NiV-infected cells (nuclei indicated by dashed lines) were detected as described above. Scale bars, 10 µm.

To corroborate the hypothesis that paracrine IFN signaling in non-infected cells is functional while autocrine IFN-signaling is blocked in NiV-infected cells because STAT1 and STAT2 are sequestered to IBs, we examined the formation and localization of phosphorylated STAT proteins in NiV-infected cell cultures. As shown in Figure 6, pSTAT1 (Figure 6A) and pSTAT2 (Figure 6C) were not detected in nuclei of NiV-infected cells. In contrast, adjacent non-infected cells showed clear nuclear pSTAT staining (Figure 6A,C, enlarged areas). This indicates that IFNs secreted from infected cells could induce STAT phosphorylation only in non-infected cells (paracrine signaling).

Even after the addition of exogenous IFN-β, no pSTAT1 or pSTAT2 was detected in nuclei of NiV-positive syncytia (Figure 6B,D, nuclei highlighted by dashed lines). This shows that infected cells did not respond in an autocrine manner to type I or III IFNs secreted from the infected cells themselves, nor to additionally added IFN. Paracrine IFN signaling in non-infected neighboring cells was, however, functional and resulted in pronounced nuclear pSTAT accumulation. This supports our idea that the observed ISG expression in NiV-infected cell cultures (Figure 5B) was mainly based on ISG upregulation in non-infected cells of the monolayer.

### 3.6. Model for NiV IB-Mediated Blockade of Autocrine IFN Signaling

**Figure 7:**
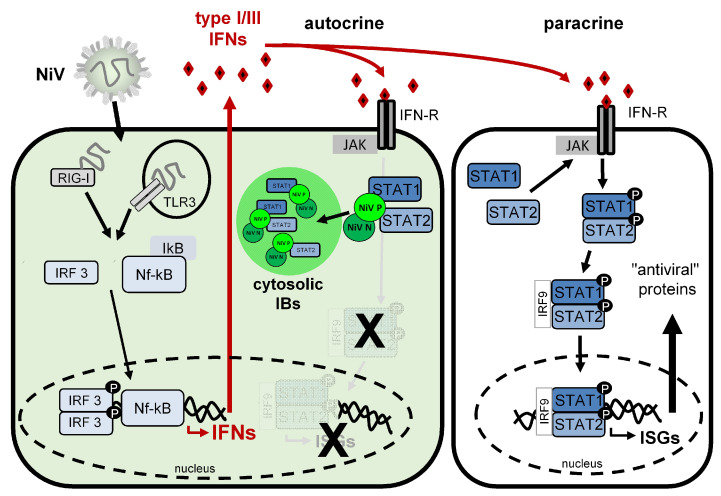
Model illustrating NiV-mediated blockade of autocrine IFN signaling by STAT sequestration to cytosolic IBs. In cells infected with NiV (cell on the left), viral RNA is recognized via cytosolic RIG-I or endosomal TLR3. This leads to the activation of IRF3 and NF-κB, which migrate to the nucleus and induce the expression of type I and type III IFNs. IFNs are secreted by infected cells and bind to their respective IFN receptors (IFN-R) on the infected cell itself or on non-infected neighboring cells, inducing either autocrine or paracrine IFN signaling. In infected cells (left), viral N and P proteins rapidly form cytosolic IBs into which STAT1 and STAT2 are recruited by the NiV P protein. Because free cytosolic STAT1 and STAT2 is lacking and cannot be phosphorylated by JAK in response to IFN receptor activation, autocrine IFN signaling cannot occur and antiviral ISG expression is prevented in infected cells. In contrast, paracrine IFN signaling is functional. In non-infected cells (cell on the right), cytosolic non-activated STAT1 and STAT2 are recruited and phosphorylated by JAK in response to IFN receptor activation. pSTAT1 and pSTAT2 dimerize and, together with IRF9, translocate to the nucleus to drive the expression of ISGs. Consequently, antiviral proteins are expressed in non-infected cells.

Overall, the results of this study provide clear evidence that the NiV P protein-mediated sequestration of inactive STAT1/STAT2 to cytosolic IBs plays a functional role in blocking autocrine IFN signaling in infected cells. As a result, IFNs secreted from these infected cells primarily activate paracrine IFN signaling and antiviral ISG responses in neighboring cells. A model illustrating these conclusions is shown in Figure 7.

## 4. Discussion

Paramyxoviruses often counter host cell innate immune responses by expressing IFN antagonistic proteins using strategies such as the degradation, disruption or inhibition of transduction factor phosphorylation to counteract antiviral gene expression. Although the specific mode of action varies between different viruses, STAT1 and STAT2 are major targets because of their essential roles in IFN signaling and ISG expression [33,34,36]. In line with this concept, the potential of NiV to efficiently replicate in host cells is linked to its ability to interfere with the cellular IFN system (reviewed in [37]). Important IFN-antagonistic proteins are the NiV P-gene-encoded nonstructural accessory proteins V and W. As they share common STAT1 and STAT2 binding domains with the full-length P protein, all three NiV proteins can individually inhibit IFN-dependent STAT signaling [38,39,42]. While the antagonistic functions of the nonstructural V and W proteins have been well characterized over the years, the functional role of the P protein in a full viral context is less defined. Although it is known that NiV P as a single protein can bind to STAT1/2 and can block IFN signaling [38,39], it is not clear how the presence of other viral proteins and changes in intracellular distribution, namely accumulation in IBs, may affect its IFN antagonistic function. With this study, we have now shown that this intracellular concentration of NiV P plays an important role because it also leads to the compartmentalization of STAT1 and STAT2. Our findings support the idea that the NiV P-mediated sequestration of STAT proteins into viral IBs reduces the pool of “free” STAT1 and STAT2, so that cytosolic STAT phosphorylation in response to IFN is limited. Consequently, pSTAT translocation into the nucleus to initiate antiviral gene expression is blocked in infected cells. This demonstrates for the first time that cytosolic NiV IBs play a functional role in IFN antagonism and suggests that blocking autocrine IFN signaling in infected cells by sequestering STAT proteins to IBs provides a mechanism for NiV to block IFN-dependent antiviral gene expression, in addition to the IFN-antagonistic functions of its accessory proteins.

### 4.1. Targeting of STAT Proteins by NiV P Gene Products

The P gene of NiV encodes three STAT-binding proteins (P, V, W), which all bind to STAT1 via the N-terminal amino acids 114–140, and to STAT2 via the region between residues 100 and 300 [39,41,46,56]. When expressed individually, physical interactions of the three proteins with STATs did not result in STAT degradation but in recruiting STAT away from activating JAK [41]. However, in transfection systems, P, V and W differed in their capacities to inhibit STAT signaling, with P being the least effective [38]. This might be explained by the fact that V and W proteins have evolved to specifically act as IFN antagonists by sequestering STATs in the cytoplasm and nucleus, respectively. In contrast, the STAT-blocking function of the P protein might only be secondary, since NiV P is primarily required for viral replication, particularly for “chaperoning” the NiV N protein, and as a cofactor of the NiV polymerase [57].

As the V and W proteins are not needed for productive NiV replication, studies with recombinant NiV with genetically knocked out accessory proteins were used to confirm their functional roles in modulating NiV pathogenesis in animal models [43,44,45]. Because none of these studies used recombinant viruses lacking all accessory proteins simultaneously, the extent to which the P protein alone can inhibit the IFN signaling in vivo remains to be determined. However, our finding that P-containing IBs in transfected cells, i.e., in the absence of accessory proteins, were sufficient to almost completely sequester steady-state STAT1/2 (Figure 2) suggests that the P protein plays a more important role in blocking STAT signaling than previously thought [38]. Because of the ability not only to complex both STATs but also to sequester them to constantly growing IBs, P-dependent STAT blockade likely contributes importantly to the antiviral countermeasures of NiV. Nevertheless, it must be assumed that, to optimally suppress antiviral gene expression, the P-mediated sequestration of STAT1 and STAT2 to IBs is accompanied and complemented by cytosolic V-mediated STAT1/2 binding and the W-mediated nuclear retention of STAT1. The fact that the V and W proteins play an important role in inhibiting IFN signaling is well established and was particularly clearly demonstrated in a study using P-mRNA editing defective NiV mutants [58]. However, the importance of the respective P, V, W-dependent STAT-blocking pathways likely varies in different infection situations and depends on multiple parameters, such as the cell type, STAT levels, time point of infection, kinetics of IB formation, extent of syncytia formation and, most importantly, the levels of P, V and W expression, which are influenced by the efficiency of P mRNA editing [53]. Infection studies with appropriate NiV mutants—for example, P editing defective NiV mutants [58]—could help to further define the individual IFN antagonistic roles of the three P gene products in different infection settings.

Now that we have shown that P-mediated STAT inhibition is spatially distinct from the V-mediated blockade, which occurs dispersed throughout the cytoplasm [53], P-mediated STAT1/2 inhibition, which, in previous models, is represented in the cytoplasm side by side with the V protein [37,41,59,60], can now be specified and assigned to IBs.

### 4.2. Blockade of STAT Complex Formation by NiV N

Aside of the NiV P gene products, NiV N was shown to inhibit IFN signaling by preventing the translocation of pSTAT1 complexes to the nucleus [61]. Interestingly, the N protein did not inhibit the phosphorylation of STAT but its complex formation in the cytoplasm. As NiV N cannot bind to STAT proteins, the underlying mechanism is still unclear but is obviously distinct from the mechanism used by the P protein, which prevents STAT phosphorylation by binding and sequestering inactive STATs to IBs. The NiV N-mediated inhibition of STAT complex formation might be an additional pathway in suppressing IFN responses. However, since pSTAT formation is not inhibited by NiV N [61], this cannot account for the absence of pSTAT observed here in cells with IBs. The conclusion that the blockade of IFN signaling that we saw in NiV N and P coexpressing cells was mediated by P is strengthened by the finding that despite the presence of NiV N, nuclear pSTAT1 accumulation was blocked only by wildtype NiV P but not by a STAT1-binding defective NiV P protein (P_G125E_) [41] (Figure S1).

### 4.3. STAT Sequestration to IBs

Although almost all pathogenic viruses express some IFN-antagonistic proteins that complex or degrade STAT1/2, there are very few reports linking STAT blockade and IBs. The most detailed studies are from two phleboviruses, severe fever with thrombocytopenia syndrome virus (SFTSV) and Guertu virus (GTV). The nonstructural proteins of these viruses mediate the formation of cytoplasmic IBs, which serve as a site for the sequestration of various innate signaling molecules, including STAT2 (but not STAT1), thereby blocking IFN induction and signaling [62,63]. Phlebovirus IBs in SFTSV- and GTV-infected cells are formed by a nonstructural viral protein, and their main function is to sequester antiviral cellular factors, which is why they are even called “IB jails”. As such, they principally differ from the cytosolic IBs found in cells infected with paramyxoviruses, or MNS in general. These are primarily involved in viral replication and contain viral nucleocapsid proteins as scaffolds. Nevertheless, there is increasing evidence that MNS IBs have additional functions besides their role in replication, and can sequester some cellular proteins [24,26]. For example, filoviruses hijack several host cell proteins to IBs, which directly support virus replication [64,65,66,67]. Furthermore, there are some reports about MNS IBs recruiting innate signaling molecules of the RIG-I/TLR3 pathways, thereby hampering RNA sensing and IFN production [68,69,70,71]. Data linking any innate signaling molecules and NiV IBs are completely lacking so far. Therefore, our discovery of STAT1 and STAT2 sequestration to IBs is the first report hinting at the involvement of cytosolic IBs in the downregulation of IFN responses in NiV-infected cells. A similar colocalization of IBs with both STAT proteins required for IFN signaling was so far only observed for one other paramyxovirus, namely measles virus (MeV) [72,73]. Because the data are still too limited to draw overall conclusions, it would be important to study other paramyxoviruses whose P proteins also accumulate in IBs and can bind STAT proteins to clarify whether paramyxovirus IBs might play a general role in antagonizing STAT-dependent IFN signaling.

## 5. Conclusions

In summary, we propose that successful NiV replication depends on multifactorial inhibition of the IFN response, with an important role for the blockade of antiviral gene expression induced by autocrine IFN signaling. This inhibition is mediated not only by STAT-binding accessory NiV V and W proteins but also by P-dependent STAT1/2 sequestration to IBs. As such, IBs not only compartmentalize viral replication but also IFN-antagonistic functions.

## Data Availability

Further information on the presented data is available on request.

## Supplementary Materials

The following supporting information can be downloaded at: https://www.mdpi.com/article/10.3390/v15020554/s1, Table S1: List of primary and secondary antibodies; Table S2: Primer pairs used for qPCR; Figure S1: Wildtype NiV P but not STAT1-binding defective NiV P_G125E_ blocks nuclear STAT1 accumulation in IFN-treated cells.
