# Supplementary material for: Nipah Virus Impairs Autocrine IFN Signaling by Sequestering STAT1 and STAT2 into Inclusion Bodies
Source: Viruses. 2023 Feb 17;15(2):554. doi: 10.3390/v15020554 (PMC9967463; doi:10.3390/v15020554)
Supplement: Supplementary file 1 [file viruses-15-00554-s001.zip › viruses-2184084-supplementary.pdf]

Nipah virus impairs autocrine IFN signaling by sequestering STAT1 and STAT2 into inclusion bodies

Nico Becker, Andrea Maisner *

Institute of Virology, Philipps University Marburg, Hans-Meerwein-Str. 2, 35043 Marburg, Germany

*   Correspondence: maisner@uni-marburg.de

Supplementary Material

In order of appearance in the manuscript

Supplementary Table 1

Table S1. List of primary and secondary antibodies.

Primary antibodies	Source	Dilution	Provider	Catalogue number	Permeabilization after 4% PFA fixation
**anti-NiV N/P (GP3)**	guinea pig	1:500	Heinz Feldmann (Hamilton, MT, USA)		0.1% Triton X-100
**anti-STAT1**	mouse	1:50	Santa Cruz	sc-464	0.1% Triton X-100
**anti-STAT2**	mouse	1:50	Santa Cruz	sc-1668	0.1% Triton X-100
**anti-pSTAT1**	rabbit	1:100	Invitrogen	MA5-15071	100% methanol
**anti-pSTAT2**	rabbit	1:50	Cell Signaling Technology	88410	100% methanol
**anti-IRF3**	rabbit	1:400	Cell Signaling Technology	11904	0.1% Triton X-100
**anti-Nf-κB**	rabbit	1:400	Cell Signaling Technology	8242	0.1% Triton X-100

Secondary antibodies	Source	Dilution	Provider	Catalogue number
**anti-mouse IgG Alexa Fluor 568**	goat	1:300	ThermoFisher	A-11031
**anti-rabbit IgG Alexa Fluor 568**	goat	1:300	ThermoFisher	A-11036
**anti-guinea pig IgG Alexa Fluor 488**	goat	1:300	ThermoFisher	A-11073

Supplementary Table 2

Table S2. Primer pairs used for qPCR.

Genes	Primer pairs	Primer sequences	Reference
**Human α-Tubulin**	hu TubA1b forward<br>hu TubA1b reverse	5′-ACCACCCTGGAGCACTCTGA-3′<br>5′-GACAGATGTCATAGATGGCCTCAT-3′	[51]
**Human IFN-β**	hu IFNβ forward<br>hu IFNβ reverse	5′-TGCCTCAAGGACAGGATGAAC-3′<br>5′-GGAACTGCTGCAGCTGCTTA-3′	[51]
**Human IFN-λ2,3**	hu IFNλ2/3 forward<br>hu IFNλ2/3 reverse	5'-GCCAAAGATGCCTTAGAAGAG-3'<br>5'-CAGAACCTTCAGCGTCAGG-3'	[51]
**Human ISG56**	hu ISG56 forward<br>hu ISG56 reverse	5′-CAGCAACCATGAGTACAAAT-3′<br>5′-AAGTGACATCTCAATTGCTC-3′	[51]
**Human OAS**	hu OAS forward<br>hu OAS reverse	5′-GCCCTGGGTCAGTTGACTGG-3′<br>5′-TGAAGCAGGTGGAGAACTCGC-3′	[51]
**Human PKR**	hu PKR forward<br>hu PKR reverse	5′-ACACTCGTCTCTGAATCATC-3′<br>5′-GAGACCATTCATAAGCAACG-3′	This study
**Human MxA**	hu MxA forward<br>hu MxA reverse	5′-ATGAGCTAATCACCCTGGAG-3′<br>5′-ATACCCAATGTCAGCAGGC-3′	This study

Supplementary Figure 1

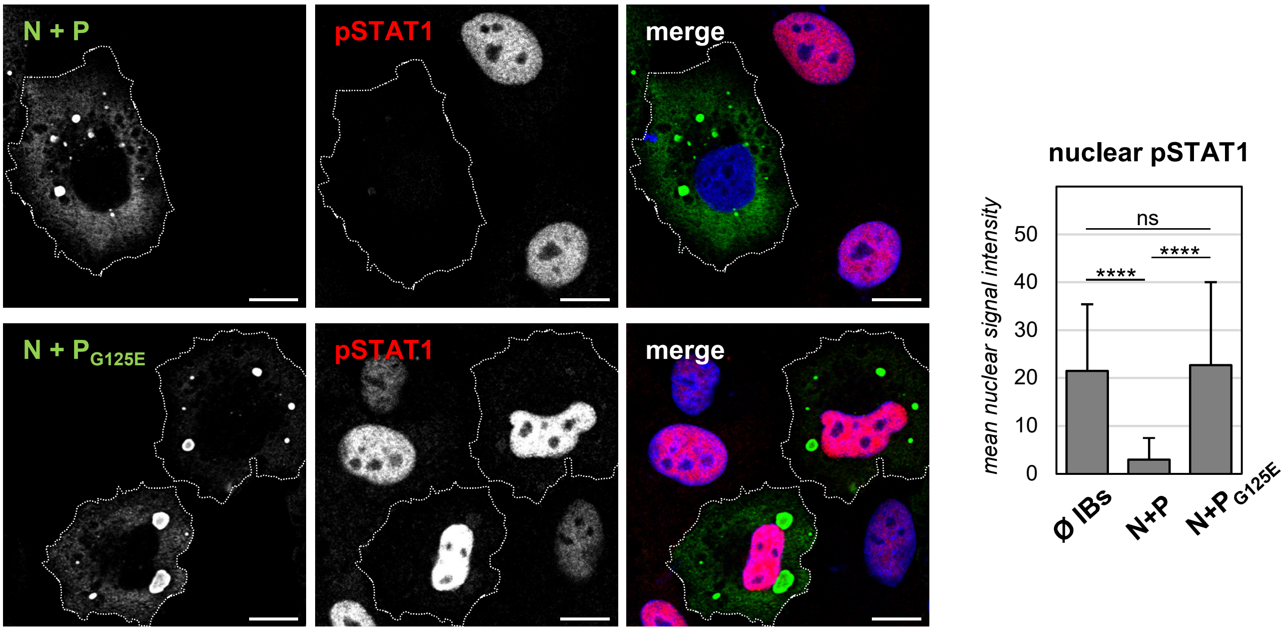

Figure S1. Wildtype NiV P but not STAT1-binding defective NiV P_{G125E} blocks nuclear pSTAT1 accumulation in IFN-treated cells.

A549 cells were transfected with plasmids encoding NiV N and NiV P (N+P) or NiV N and NiV P_{G125E} (N+P_{G125E}), a mutant P protein which is known to be STAT1-binding defective [41].

After 22 h, IBs had formed and cells were treated with 1000 U/ml of human IFN-β for 40 min and fixed with 4% PFA. After permeabilization with methanol at -20°C , endogenous pSTAT1 was stained with specific antibodies (red). IBs were detected with a NiV N/P specific antiserum (green). Nuclei were counterstained with DAPI (blue). Greyscale single-channel images and colorized merged images are shown. The dashed lines indicate IB-containing cells. Scale bars, 10 µm.

Quantification (right panel): To quantify the nuclear pSTAT1 fluorescence in IFN-treated cells, the signal intensities of pSTAT1 in the nucleus of cells without IBs (Ø IBs), cells with IBs containing wildtype P (N+P) and cells with IBs containing STAT1-binding defective P_{G125E} (N+P_{G125E}) were analyzed (> 800 cells in total). The mean nuclear signal intensity of pSTAT1 is shown. Error bars indicate standard deviation (SD); ****, $p < 0.0001$; ns, not significant.
